# Effects of low-dose X-ray medical diagnostics on female gonads: Insights from large animal oocytes and human ovaries as complementary models

**DOI:** 10.1371/journal.pone.0253536

**Published:** 2021-06-24

**Authors:** Nicola Antonio Martino, Rossella Vicenti, Maria Macciocca, Renato Seracchioli, Giuseppina Marzano, Antonella Mastrorocco, Giovanni Michele Lacalandra, Michele Tomaiuolo, Giuliana Marchesani, Eugenio Antonio Chiaravalle, Francesca Gioia Klinger, Serena Marcozzi, Raffaella Fabbri, Maria Elena Dell’Aquila

**Affiliations:** 1 Department of Biosciences, Biotechnologies & Biopharmaceutics, University of Bari Aldo Moro, Valenzano, Bari, Italy; 2 Gynecology and Physiopathology of Human Reproduction Unit, Department of Medical and Surgical Sciences, University of Bologna S. Orsola-Malpighi Hospital of Bologna, Bologna, Italy; 3 Department of Veterinary Medicine, University of Bari Aldo Moro, Valenzano, Bari, Italy; 4 Istituto Zooprofilattico Sperimentale di Puglia e Basilicata, Foggia, Italy; 5 Department of Biomedicine and Prevention, Section of Histology and Embryology, University of Rome Tor Vergata, Rome, Italy; Infertility Unit, ASST Lariana, ITALY

## Abstract

Diagnostic imaging has significantly grown over the last thirty years as indispensable support for diagnostic, prognostic, therapeutic and monitoring procedures of human diseases. This study explored the effects of low-dose X-ray medical diagnostics exposure on female fertility. To aim this, cumulus-oocyte complexes (COCs) recovered from the ovaries of juvenile sheep and human ovaries were used as complementary models for in vitro studies. In the sheep model, the effects of low-dose X-rays on oocyte viability and developmental competence were evaluated. In human ovaries originated from two age group (21–25 and 33–36 years old) subjects with gender dysphoria, X-rays effects on tissue morphology, follicular density and expression of apoptosis-related (NOXA, PUMA, Bcl2, Bak, γH2AX) and cell cycle-related genes (p21 and ki67) were investigated. It was noted that in sheep, the minimum dose of 10 mGy did not influence most of examined parameters at oocyte and embryo levels, whereas 50 and 100 mGy X-ray exposure reduced oocyte bioenergetic/oxidative activity but without any visible effects on oocyte and embryo development. In addition, blastocyst bioenergetic/oxidative status was reduced with all used doses. Overall data on human ovaries showed that low-dose X-rays, similarly as in sheep, did not alter any of examined parameters. However, in women belonging to the 33–36 year group, significantly reduced follicular density was observed after exposure to 50 and 100 mGy, and increased NOXA and Bax expression after exposure at 50 mGy. In conclusion, used low-doses of X-ray exposure, which resemble doses used in medical diagnostics, produce weak damaging effects on female fertility with increased susceptibility in advanced age.

## Introduction

In the last thirty years, diagnostic imaging had a considerable development, thanks to significant innovation of image detectors and computer science, becoming an indispensable support for diagnosis, prognosis and monitoring of diseases, and the implementation of interventional diagnostic and therapeutic procedures [1,2]. Today, the medical use of ionizing radiation is the main exposure source of artificial radiation for the population, and it is constantly increasing in all countries with modern health care systems. X-ray imaging comprises 80 to 90 percent of all imaging procedures typically divided into "conventional" and "contrast" techniques. However, frequency of contrast studies decreased from 1970 to 1980, as competing techniques such as endoscopy, ultrasound and computed tomography (CT) became available [3].

Mettler et al. [4] reported an increase in the annual effective dose per capita (data collected from 1980–2006) from 0.54 millisieverts (mSv; 1 Sv ≅ 1 gray (Gy); [5]) to about 3.0 mSv, due to the use of CT and nuclear medicine techniques. An updated version of this report (data collected from 2006 to 2016) showed that the effective dose from diagnostic and interventional medical procedures is estimated at 2.3 mSv [6]. Interestingly, comparisons of radiation doses from diagnostic examinations with average natural back-ground radiation, show that the effective doses for normal-sized adults undergoing body CT and chest x-ray are approximately 3.5 mSv and 0.1 mSv, respectively, which is the equivalent to 1 year and 10 days of natural background radiation humans are exposed as part of our normal lives, respectively, [7–9]. The radiation doses of some CT and nuclear medicine studies fall in the range between 10 and 100 mSv. A single CT of the abdomen may produce a dose of around 10 mSv, and patients who undergo multiple CTs or a single multiphasic CT fall into this dose range [7].

The biological response to radiations varies between tissues and organs, and gonads are considered highly sensitive to radiation damage [10–12]. Ovaries contain a limited number of resting oocytes, which become reduced with increasing age and cannot be regenerated over a lifetime. As age progresses, subsequent ovulations and atresia determine progressive depletion of the follicular population [13]. When the follicle reserve is exhausted, usually in women around fifties, menopause occurs as a direct consequence of ovarian senescence [5]. Therefore, any factor that can damage the ovary unavoidably can accelerate the physiological aging and, consequently, the onset of ovarian failure, in terms of loss of primordial follicle reservoir, impaired fertility and premature menopause.

Most of information on X-ray radiation effects on the ovary comes from studies on women receiving pelvic irradiation during treatments of malignant tumours of the abdomen, craniospinal irradiation for the treatment of brain tumours or total body irradiation (TBI) before bone marrow transplantation [14–16]. Ovarian failure has been reported in 90% of patients after TBI (10–15.75 Gy; [14]) and 97% of females treated with abdominal irradiation (20–30 Gy) during childhood [17]. Radiotherapy causes damage to ovarian blood vessels, as well as initiation of cortical fibrosis and follicular apoptosis [18]. Studies performed in different species, including guinea pigs, rodents, monkeys and humans, show that the radiosensitivity of the oocytes varies according to the follicle/oocyte stage and species [5]. In mentioned species, oocytes near ovulation show the highest susceptibility to radiation induced mutational events. In fact, in women, primordial oocytes are more resistant to the effects of radiation than oocytes in growing follicles. The median lethal dose (LD_50_) for the human oocyte has been estimated to be <2 Gy [15]. While clinical evidences on effects of exposure to high-dose radiation therapy on female fertility have been reported, data on the effects of low-dose exposure on the ovary and the magnitude of risk of radiological diagnostic are still missing.

The aims of the present study were to investigate the effects of low-dose X-ray radiations (≤ 100 mGy) on ovarian viability and functional competence. To achieve this aim, ovine cumulus-oocyte complexes (COCs) and human ovarian tissues were used as complementary in vitro models. The effects of low-dose X-ray radiations have been estimated on: 1) ovine COCs, analysed for cumulus cell apoptosis, oocyte nuclear maturation, as well as bioenergetic/oxidative status, embryo cleavage, blastocyst formation rate and quality; and 2) human ovarian tissues, analysed for morphology and expression of DNA damage marker γH2AX, apoptosis-related genes, and proteins and cell cycle-related genes.

## Materials and methods

### Chemicals

All chemicals for in vitro cultures and analyses, unless otherwise indicated, were purchased from Sigma-Aldrich (Milan, Italy) and Gibco® (Life Technologies, Paisley, UK).

#### Cumulus-oocyte complex (COC) collection and holding

Sheep ovaries were recovered at a local slaughterhouse (Fin. Sud Import s.r.l.; Conversano, Bari, Italy) from juvenile ewes (age under 6 months) subjected to routine veterinary inspection (Council Directive 89/556/ECC and subsequent modifications), and transported to the laboratory at room temperature within 4 h of slaughtering. For COC retrieval, ovaries were processed by the slicing procedure [19]. COCs were placed in glass vials with 1 mL of holding medium (HM) composed by 40% (v/v) TCM-199 with Earle’s salts, 40%(v/v) TCM-199 with Hanks’ salts and 20% (v/v) fetal calf serum (FCS) with 25 mg/mL gentamicin [20]. Holding medium was used for COC transport, irradiation and overnight maintaining before IVM.

#### COC and ovarian tissue X-ray irradiation

Sample irradiation was carried out at CRN-Radioactivity of IZS-PB (Foggia, Italy) with the RS-2400 biological irradiator, consisting of a cylindrical X-ray tube with a tungsten cathode and a golden target. In this irradiator, the maximum allowed anode current is 45 mA and the voltage can be varied up to 150 kV, enabling variable quality of the irradiation beam. To obtain the required dose levels, a lead shield was designed in order to limit the intensity of the beam. The sizing of the lead screen was initially carried out with a PENELOPE-based system for the automated Monte Carlo simulation of electron and photon transport [21] and then dosimetry measurements with thermoluminescence dosimeters were carried out. The lead thickness of 1 mm gave satisfactory results for the proposed purposes. The dosimetry control of the irradiation process was carried out with TLD-700 thermoluminescence dosimeters. Samples were exposed with a constant dose/rate of 0.6 mGy/s. The irradiation times were 50s for 10 mGy, 83s for 50 mGy and 166s for 100 mGy. For the positive control, a dose/rate of 75 mGy/s was used for 33s. Irradiated and control samples were transported back to the laboratory and kept overnight at room temperature in HM. Cryopreserved ovarian tissue samples, while included in cryovials, were inserted into Petri dishes containing liquid nitrogen, placed in an aluminium sample holder shielded with a lead plate suitably sized to impart to the samples the low doses of irradiation. Similarly, ovine COCs located in glass vials with 1 mL of HM, were placed in the aluminium sample holder as previously described. The sizing of the shielding was carried out with the aid of Monte Carlo simulations and subsequent experimental verification with thermoluminescence dosimeters.

#### In vitro maturation

On the day after irradiation, in vitro maturation (IVM) was performed in TCM-199 medium with Earle’s salts, buffered with 5.87 mM HEPES and 33.09 mM sodium bicarbonate, supplemented with 0.1 g/L L-glutamine, 2.27 mM sodium pyruvate, calcium lactate pentahydrate (1.62 mM Ca^2+^, 3.9 mM Lactate), 50 μg/mL gentamicin, 20% (v/v) FCS, 10 μg/mL ovine follicle stimulating hormone (FSH), 20 μg/mL ovine luteinizing hormone (LH) and 1 μg/mL 17β estradiol [22]. COCs were placed in 400 μL of IVM culture medium/well of a four-well dish (Nunc Intermed, Roskilde, Denmark), covered with pre-equilibrated lightweight paraffin oil and incubated for 24 hours at 38.5°C, under 5% CO_2_ conditions. For each experimental condition, 20–25 COCs were analysed in each replicate and a minimum number of three replicates were performed. After IVM, cumulus cell removal was performed by incubation in TCM-199 with 20% FCS containing 80 IU hyaluronidase/mL and aspiration in and out of finely drawn glass pipettes. COCs destined to microscopy analysis or those destined to IVF and subsequent in vitro embryo culture underwent total or partial cumulus cell removal, respectively.

#### In vitro fertilization (IVF) and in vitro embryo culture

In vitro fertilization was performed in Synthetic Oviductal Fluid (SOF) medium supplemented with 2% oestrous sheep serum (OSS) and 1 μg/mL heparin, as described by Martino et al. [19]. Briefly, frozen-thawed spermatozoa were selected by the swim-up technique and used at the final concentration of 1.5 x 10^6^ spermatozoa/ml. Oocytes and sperm cells were incubated for 22 h at 38.5°C and under a 5% CO_2_, 5% O_2_ and 90% N_2_ atmosphere in four-well dishes. After IVF, presumptive zygotes were partially freed of cumulus cells and cultured for 7 days in four-well dishes in SOF medium with essential and non-essential amino acids at oviductal concentrations [23] and 0.4% BSA under mineral oil, in maximum humidified atmosphere with 5% CO_2_, 5% O_2_ and 90% N_2_ at 38.5°C [19]. Embryo development was followed by conventional morphology assessment, under phase contrast microscopy, and confirmed at day 7 (d7) by observing embryo blastomere nuclear chromatin under epifluorescence microscopy after Hoechst staining. Blastocyst formation was assessed at d7 and blastocysts were classified according to expansion and hatching status [19] as: early blastocyst (normal blastocyst with a blastocoel equal or up to half of the embryo volume), expanded blastocyst (blastocyst with a blastocoel greater than half of the embryo volume) and hatching or already hatched blastocyst.

#### Assessment of cumulus cell apoptosis by TUNEL assay

Cumulus cells, grouped according to each experimental condition, were collected from in vitro cultured COCs, suspended in TCM-199 with Hanks’ salts with 20% FCS and centrifuged at 300 x g for 5 min. The resulting pellet was used for assessing the apoptotic status by Terminal Deoxynucleotidyl Transferase-mediated dUTP Nick-End Labeling (TUNEL) assay (Click-iT® Plus TUNEL Assay for in situ apoptosis detection with Alexa Fluor® dyes, Molecular Probes Life Technology, code: C10617; [22]). The staining procedure was performed following the manufacturer’s instructions. Briefly, CCs were fixed in 4% paraformaldehyde in phosphate buffered saline solution (PBS) for 15 min at room temperature. CCs were washed three times in PBS and then permeabilized with 0.5% Triton X‐100 for 20 min. CCs were washed with deionized water before labeling. CCs were placed in 50 μl drops of TUNEL reagent and incubated in the dark for 1 h at 37°C in a humidified chamber. After incubation, CCs were washed three times with 3% Bovine Serum Albumin (BSA) in PBS. Total cell nuclei were stained with 2.5 μg/ml Hoechst 33258 in 3:1 (vol/vol) glycerol/PBS, mounted on microscope slides, covered with cover‐up micro slides, sealed with nail polish and kept at 4°C in the dark until observation. CCs were observed under an E‐600 Nikon fluorescent microscope equipped with a 365 nm excitation filter. Apoptosis was determined as the percentage of labeled cells (TUNEL positive) to the total cell number (Hoechst 33258) [22]. For each condition, a minimum of 800–1000 randomly chosen CCs was examined.

#### Oocyte and blastocyst mitochondria and ROS staining

In order to detect and localize mitochondria, oocytes and blastocyst were washed three times in PBS with 3% BSA and incubated in the same medium containing 280 nM MitoTracker Orange CMTM Ros (Molecular Probes) for 30 min at 38.5°C, under 5% CO_2_ [19]. After incubation with MitoTracker, in order to detect intracellular sources of ROS, oocytes and blastocyst were washed in PBS with 0.3% BSA and incubated for 15 min at 38.5°C under 5% CO_2_ in the same medium containing 10 μM 2’,7’- dichlorodihydrofluorescein diacetate (H_2_DCF-DA). After incubations, oocytes and blastocysts were washed in PBS and fixed overnight at 4°C in 2% paraformaldehyde solution in PBS [19].

#### Nuclear chromatin evaluation of oocytes and embryos

After fixation, oocytes and embryos were stained with 2.5 μg/mL Hoechst 33258 in 3:1 (v/v) glycerol/PBS and mounted on microscope slides with coverslips, sealed with nail polish and kept at 4°C in the dark until observation. Slides were examined under an epifluorescence microscope (Nikon Eclipse 600; ×400 magnification) equipped with a B‐2A (346 nm excitation/ 460 nm emission) filter. Oocytes were evaluated in relation to their meiotic stage and classified as germinal vesicle (GV), metaphase to telophase I (MI to TI), MII with the 1st polar body extruded, or as degenerated [19]. Early embryos were evaluated according to their number of nuclei. They were indicated as morulae if they contained more than 32 cells but did not have an organized outer layer of cells, and as blastocysts, if they contained more than 64 cells and had initiate the organization of outer presumptive trophoblast cells [22].

#### Assessment of mitochondrial distribution pattern and intracellular ROS localization

Oocytes at the MII stage and blastocysts were observed at x600 magnification in oil immersion with a Nikon C1/TE2000‐U laser scanning confocal microscope. A helium/neon laser ray at 543 nm and the G‐2A filter (551 nm excitation and 576 nm emission) were used to point out the MitoTracker Orange CMTMRos. An argon ion laser ray at 488 nm and the B‐2A filter (495 nm excitation and 519 nm emission) were used to point out the DCF. Scanning was conducted with 25 optical series from the top to the bottom of the oocytes and embryos with a step size of 0.45 μm to allow three dimensional distribution analysis. The mitochondrial distribution pattern was evaluated on the basis of previous studies [24–26]. Evaluation was performed according to the followed criteria: (a) a homogeneous distribution of small mitochondria aggregates throughout the cytoplasm was considered as an indication of low energy cytoplasmic condition; (b) perinuclear (with mitochondria more concentrated in the oocyte hemisphere where the meiotic spindle is located) and subplasmalemmal (forming large granules in the cortical region) was considered as characteristic of healthy cytoplasmic condition (P/S); and (c) an irregular distribution of mitochondria, with large mitochondrial clusters, were classified as abnormal. Concerning intracellular ROS localization, oocytes and embryos with intracellular ROS diffused throughout the cytoplasm together with areas/sites of mitochondria/ROS overlapping were considered healthy [24–26].

#### Quantification of MitoTracker Orange and DCF fluorescence intensity

In each individual MII oocyte and blastocyst, MitoTracker Orange and DCF fluorescence intensities were measured at the equatorial plane for the oocytes and on all 25 focal planes for blastocysts, at the excitation/emission as described above, with the aid of the EZ-C1 Gold Version 3.70 image analysis software platform for Nikon C1 confocal microscope [19,22]. For each focal plane, a circle area was drawn in order to measure only the area including cell cytoplasm. Colocalization analysis of mitochondria and ROS was performed, as previously reported, with the same software. Degree of colocalization was reported as the overlap degree between MitoTraker Orange and DCF fluorescence signals [19,22]. Mitochondria/ROS co-localization is reported as a biomarker of healthy oocytes and embryos [19,25–27].

#### Collection of human ovaries

The ovarian tissue was collected from six subjects (mean age ± SD: 29 ± 6.42) with gender dysphoria, who underwent bilateral hysteroannessiectomy at the Gynecology and Physiopathology Reproductive Unit of S. Orsola-Malpighi Hospital of Bologna, Italy. At the time of ovarian tissue collection, the subjects were treated with testosterone not more than one year. Serum levels of Anti-Müllerian hormone (AMH) were normal. All subjects signed written informed consent to voluntarily donate the ovarian tissue for research (approval of the Ethics Committee: protocol n° 61/2007 / O / Tess). This consent was independent of being approved to undergo bilateral hysteroannessiectomy. Patients were divided by age into two groups: 21–25 years (patients N°1, N°2 and N°3) and 3–36 years (patients N°4, N°5 and N°6).

#### Ovarian tissue cryopreservation

Ovarian tissues, obtained by laparoscopy, were immediately transferred to the laboratory in Dulbecco’s phosphate-buffered saline (DPBS) supplemented with 10% human serum (HS; provided by the Transfusion Center of S.Orsola-Malpighi Hospital) at 4°C. The ovarian medulla was removed using a surgical scissor and the cortical tissue was dissected in strips (length 1 cm × width 2 mm × height 1 mm) and cryopreserved by slow freezing [28].

#### Thawing and culturing of irradiated cryopreserved human ovarian tissue

Irradiated cryopreserved samples and CTRL cryopreserved samples were thawed using a rapid thawing protocol [29]. After thawing, for each subject and experimental condition (CTRL, 50 mGy and 100 mGy), the cortical strips were cut into 2mm x 2mm x 1mm fragments and cultured at 37° C for 24 hours and 6% CO_2_, in order to allow the tissue reactivation after thawing. The culture medium was composed of α-Minimum-Essential-Medium (α-MEM), antibiotics, Insulin-Transferrin-Selenium (ITS) 1X, N-acetylcysteine (NAC) 25 mM, Insulin growth factor-II (IGFII) 0.02 μM and 40% human serum.

#### Total RNA extraction and cDNA synthesis

RNA was extracted from ovarian fragments using TRIzol reagent (Invitrogen). Briefly, 0.5 mL of TRIzol were add per 25–50 mg of tissue. Extraction steps were performed according to TRIzol manufacturer’s instructions and the extracted RNA was quantified by NanoDrop ND-1000 Spectrophotometer (Thermo Fisher Scientific). The first strand cDNA was synthesized from 500 μg of RNA template by TransScript One-Step gDNA Removal and cDNA Synthesis SuperMix kit (TransGene Biotech) in a 20 μL reaction.

#### Real-Time PCR (qRT-PCR)

Reverse-transcribed cDNA was used for Real Time Polymerase Chain Reaction (qPCR) using iTaq Universal SYBR Green Supermix (Biorad). The mRNA expression of the following set of genes was assessed: cyclin-dependent kinase inhibitor 1 (p21), cell proliferation markers Ki-67 (ki67), phorbol-12-myristate-13-acetate-induced protein 1 (NOXA), p53-upregulated modulator of apoptosis (PUMA), B-cell lymphoma 2 (Bcl2), BCL2-associated X protein (Bax). The Glyceraldehyde-3-Phosphate Dehydrogenase (GAPDH) was used as internal reference gene. Specific forward (F) and reverse (R) primers are listed in Table 1. Primers were used at a concentration of 0.5 μM and the reaction performed on a 7300 Real-Time PCR System (Applied Biosystems). Data from the reaction were collected and analyzed using the 2^-ΔΔCt^ method. Relative gene expression analysis was performed by relating the signal of the treated group to that of CTRL.

**Table 1:** Primer sequences of analysed genes.

Gene	Forward primer	Reverse primer
**GAPDH**	5’-TCGGAGTCAACGGATTTGGT-3’	5’-GAATTTGCCATGGGTGGAAT-3’
**p21**	5’-GGCAGACCAGCATGACAGATT-3’	5’-GCGGATTAGGGCTTCCTCTT-3’
**ki67**	5’-GCCCCAACCAAAAGAAAGTCT-3’	5’-AGCTTTGTGCCTTCACTTCCA-3’
**NOXA**	5’-GGCCTGCGGTTCAAGCT-3’	5’-GCCGACGCCACATTGTG-3’
**PUMA**	5’-ACGACCTCAACGCACAGTACGA-3’	5’-CCTAATTGGGCTCCATCTCGGG-3’
**Bax**	5’-TCAGGATGCGTCCACCAAGAAG-3’	5’-TGTGTCCACGGCGGCAATCATC-3’

#### Western blot

Samples were lysed in RIPA buffer, containing 50 mM Tris-HCl pH 7.4, 150 mM NaCl, 0.5% NP-40, 5 mM EDTA, 0.5% sodium deoxycholate, 1 mM PMSF, 1mM sodium o-vanadate and protease and phosphatase inhibitors. Samples were homogenized on ice by ultrasonic homogenization and protein concentration determined by Bradford assay. Proteins (about 40 μg) were resolved on SDS‐polyacrylamide gel and transferred to a PVDF transfer membrane (GE Healthcare). Blots were blocked in 5% non-fat dry milk in PBS-T (0.05% Tween 20 in PBS) for 1h at room temperature and then incubated with primary antibodies (in 1% milk in PBS‐T) O.N. at 4°C: GAPDH (sc-32233 SantaCruz Biotech, 1:2000 dilution); anti-γH2AX (05–636 Millipore, 1:1000 dilution); Bcl-2 (sc-509 SantaCruz Biotech, 1:500 dilution); Bak (sc-832 SantaCruz Biotech, 1:500 dilution). Membranes were, then, incubated with HRP-conjugated secondary antibody for 1 h at room temperature and signals detected by peroxidase reaction using Clarity Western ECL Substrate (Biorad). Immunoblots were quantitatively evaluated using ImageJ software (NIH).

#### Histology

Ovarian tissue samples were fixed in 4% formaldehyde solution at 4°C for 48 hours. After alcohol dehydration, samples were embedded in paraffin blocks for light microscopy and sectioned (5 μm thickness) following standard histological procedures. After deparaffination and hydration, tissues were serial-sectioned and stained with haematoxylin and eosin. Sections of ovarian tissue were observed under a ×10 magnification microscope to detect artefacts and then observed at ×25 to assess developmental follicle stage, follicle preservation and stroma integrity. Serial follicle count was performed over the entire biopsy, every five sections, and, to avoid double counting, for any type of follicle, only oocytes with a visible nucleus were considered. Classification of follicles was performed according to Gougeon classification [30]; briefly, follicles were staged as: (a) primordial, when the oocyte was surrounded by a partial or a complete layer of flattened granulosa cells (GCs); (b) primary, when the oocyte was surrounded by a single layer of cuboidal GCs, and (c) secondary, when the oocyte was surrounded by more than one layer of cuboidal GCs. The total number of primordial, primary and secondary follicles per biopsy was counted in a blind fashion by two different operators with a Leitz Diaplan light microscope equipped with CCD JVC video camera (Leitz Diaplan, Wetzlar, Germany) and Image ProPlus software (MediaCybernetics, Rockville, USA). Data were reported as follicular density (referred to follicle number/mm^3^) and relative follicular density % (referred to the percentage of Treated follicle number/CTRL follicle number), necessarily to normalize acquired data for the sample volume. Briefly, (a) follicular density was calculated by dividing the total number of follicles counted by the volume of the tissue analysed, and (b) tissue volume was calculated as the total area of all sections analysed multiplied by five, the interval of analysis, and then for the thickness of sections (0.5 μm), obtaining the volume of the biopsy expressed as mm^3^. Relative follicular density % was calculated as the percentage of the Treated follicular density/CTRL follicular density.

#### Statistical analysis

The proportions of oocytes showing the different chromatin configurations and mitochondrial distribution patterns were compared among groups by Chi-square test with the Yates’ correction. The percentages of apoptotic cumulus cells and the proportion of cleaved embryos and blastocysts were compared by Chi-square test without the Yates’ correction. Mitochondria and ROS raw values of fluorescence intensities and overlap coefficient and blastocyst number of nuclei were compared by one-way ANOVA followed by Dunnett’s post hoc test. Data from human ovarian tissues, were analysed with GraphPad Prism (software version 7.0, San Diego, CA). Results were represented as mean ± SEM and *P* value was determined by one-way ANOVA and Bonferroni post-analyses. Differences with P <0.05 were considered to be statistically significant.

#### Experimental design

*Experiment 1 Effects of low-dose X-rays on COCs viability*, *maturation and bioenergetics*. The first step of the present study was to identify the range of low-dose X-ray radiations affecting COC viability and functionality. Experiments were performed in the juvenile sheep as large animal model. Because of the need to foresee round-trip transfers to the irradiation treatment site and to allow correct subsequent IVM scheduling, COCs underwent holding treatment [20], which allows to efficiently keep overnight oocytes without adversely affecting their meiotic and developmental competence. After placement in HM and transport to the irradiation unit, COCs were exposed to 0, 10, 50 or 100 mGy X-rays. The higher dose of 2500 mGy was used as positive control of damage, as corresponding to a very high exposure dose (equivalent to approximately 250 CT). After exposure, COCs continued to be held overnight in HM and day after underwent IVM. After IVM, CCs underwent TUNEL assay, whereas oocytes were analyzed for nuclear maturation rate and cytoplasmic bioenergetic/oxidative status.

*Experiment 2 Effects of low-dose X-rays on embryo developmental and quality*. A subsequent step of the study was performed to determine the effects of low-dose X-ray radiations on oocyte developmental competence. In this experiment, COC retrieval, holding, transport, X-ray irradiation and IVM were performed as described in experiment 1. After IVM, ovine COCs underwent IVF and in vitro embryo culture up to the blastocyst stage. Effects of X-ray radiations on blastocyst quality were assessed as long-term carryover effects.

*Experiment 3 Effects of low-dose X-rays on human ovaries tissue morphology and functionality*. The final step of the study was to determine the effects of low-dose X-ray radiations on human ovarian tissue morphology and gene expression. Ovarian tissue samples were transported under liquid nitrogen to the irradiation unit. For each subject, two cryopreserved ovarian tissue samples were irradiated at 50 mGy and 100 mGy. Non-irradiated cryopreserved ovarian tissue from each subject was used as a control (CTRL). After irradiation, tissue samples were thawed, in vitro cultured for 24 hours and then processed for the following analyses: histology (to evaluate the morphological features of follicles and stroma), qPCR (to evaluate the expression of transcripts involved in apoptosis [NOXA, PUMA, Bax] and cell cycle arrest [p21, ki67]); and western blot (to evaluate the expression of proteins involved in apoptotic pathways [Bcl2, Bak, γH2AX]).

## Results

### Experiment 1: Low-dose X-ray radiations affect oocyte mitochondrial function

**Fig 1:**
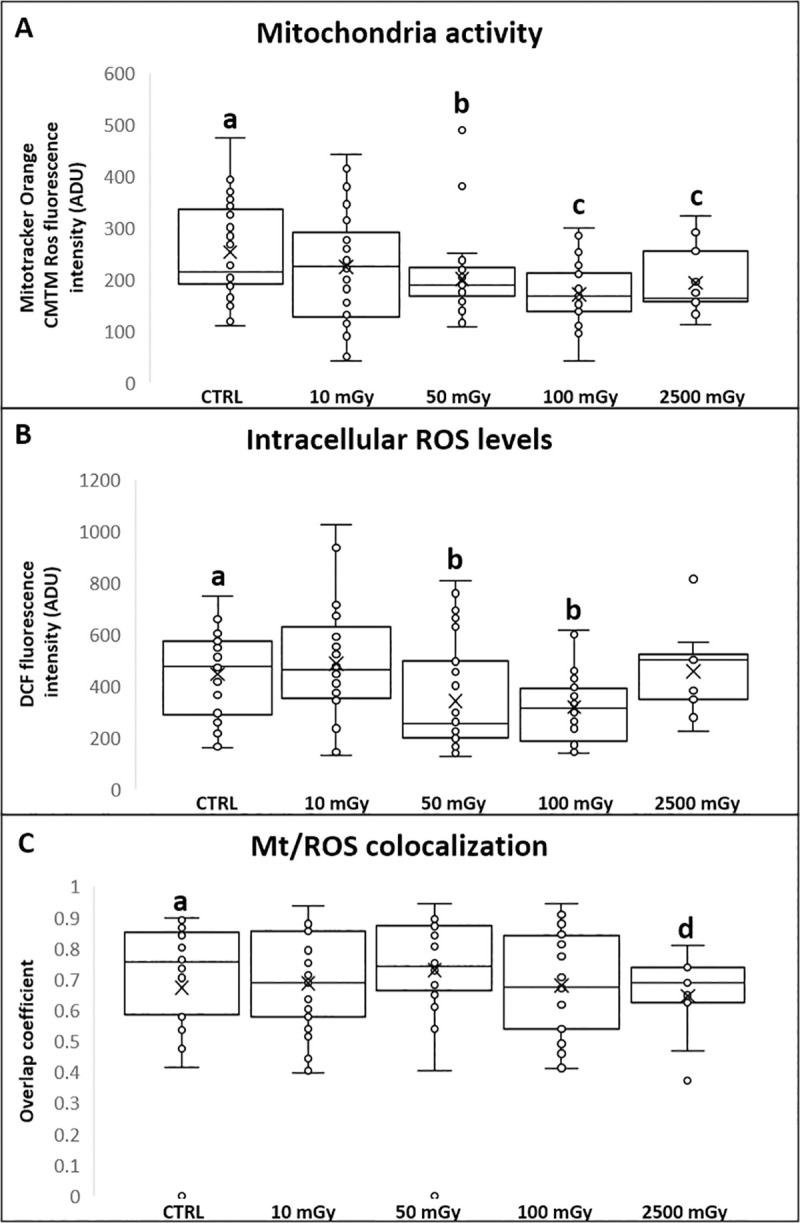
Effects of low-dose X-ray radiations before IVM on bioenergetic/oxidative status of matured oocytes. Mitochondria (mt) activity (panel A) and intracellular ROS levels (panel B) are expressed in arbitrary densitometric units (ADU) as means ± SD of MitoTracker Orange CMTMRos and DCF fluorescence intensity. Mitochondria/ROS colocalization (panel C) is expressed as means ± SD of overlap coefficient. One-way ANOVA followed by Dunnett’s post hoc test: a,b = P<0.05, a,c = P<0.01, a,d = P<0.001.

**Fig 2:**
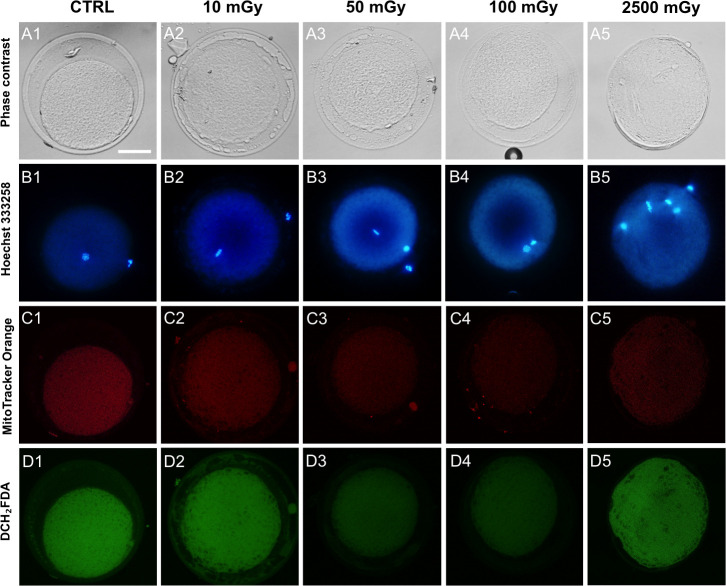
Representative micrographs of oocytes exposed to low-dose X-ray radiations and examined for their nuclear chromatin and bioenergetic/oxidative potential. Representative images of a control oocyte (column 1) and oocytes exposed to 10–2500 mGy (column 2–5) before IVM. Corresponding phase contrast images showing cell morphology (line A), epifluorescence images showing nuclear chromatin configuration (line B) and confocal images showing mitochondria (line C) and intracellular ROS (line D). Confocal images were taken at oocyte equatorial plane. Scale bar represents 40 μm.

**Fig 3:**
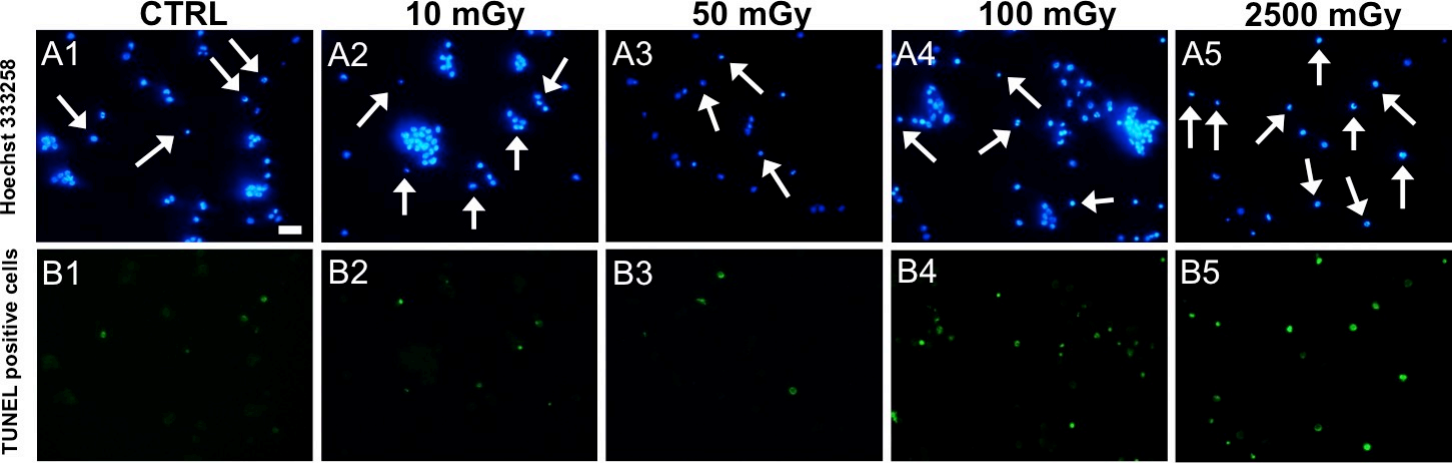
Effects of low-dose of X-ray radiations on cumulus cells apoptosis. Representative images of cumulus cells assessed for apoptotic index by TUNEL assay after COC exposure to X-ray radiations before IVM. Line A: nuclei stained with Hoechst 333258 (blue). Line B: TUNEL-positive nuclei stained in green. Scale bar represents 20 μm.

No significant effects of low-dose X-ray radiations were observed on cumulus cells apoptotic index and oocyte maturation rate (P˃0.05; Table 2). No effects were noticed on the percentages of oocytes which did not resume meiosis (GV stage) or those found at intermediate meiotic stages (MI to TI) or showing abnormal chromatin configurations. In order to explore the hypothesis whether X-rays may induce damaging effects on oocyte cytoplasmic quality, those oocytes found at the metaphase II stage were analysed for their bioenergetic/oxidative status. Mitochondrial distribution pattern did not vary after exposing COCs at low doses as the majority of oocytes showed healthy, perinuclear and subcortical, cytoplasmic distribution of mitochondria (Table 2). Remarkably, samples exposed to the positive control doses were significantly affected only for cumulus cells apoptosis but their meiotic potential and mitochondrial pattern were not affected. On the other hand, exposure to low-dose X-rays significantly reduced mitochondrial activity (P<0.05 and P<0.01, for 50 and 100 mGy respectively; Fig 1, panel A) and ROS levels (P<0.05, for 50 and 100 mGy; Fig 1, panel B) whereas mitochondria/ROS co-localization was not affected (Fig 1, Panel C). Exposure to 2500 mGy significantly reduced two quantitative bioenergetic parameters, such as mitochondrial activity (P<0.01) and mt/ROS colocalization (P<0.001) whereas ROS levels was not changed (Fig 1). Notably, exposure to the lowest dose of 10 mGy, did not result in any significant difference in all oocyte evaluated parameters. Representative micrographs of COCs exposed to low-dose X-ray radiations and examined for oocyte maturation and bioenergetic parameters, and cumulus cells apoptosis are shown in Figs 2 and 3, respectively. In exposed samples, progressive reduction of fluorescence intensity is evident for both mitochondria- and ROS-specific probes. In positive controls, a higher density of apoptotic cells is evident, as well as reduced mitochondrial activity.

**Table 2:** Effects of cumulus-oocyte complex exposure to low-dose X-ray radiations before IVM on cumulus cell apoptosis, oocyte chromatin configuration and mitochondria pattern.

X-ray dose (mGy)	N° (%) of apoptotic/examined cells	N° of analysed oocytes	Nuclear chromatin configuration N° (%)				N° (%) of MII oocytes with healthy mitochondria pattern
			GV	MI to TI	MII+PB	Abn	
**0**	95/1011 (9.39) a	55	8 (14.5)	7 (12.7)	35 (63.6)	5 (9.1)	21/34 (61.8)
**10**	48/616 (7.79)	48	6 (12.5)	6 (12.5)	32 (66.7)	4 (8.3)	14/30 (46.7)
**50**	79/881 (8.96)	56	9 (16.1)	6 (10.7)	40 (71.4)	1 (1.8)	17/36 (47.2)
**100**	101/950 (10.63)	61	8 (13.1)	10 (16.4)	37 (60.6)	6 (9.8)	18/36 (50)
**2500**	238/605 (39.33) b	68	8 (11.8)	13 (19.1)	44 (64.7)	3 (4.4)	25/38 (66)

Chi-square test: a,b P<0.001. For each experimental condition, three replicates were performed with 20–25 COCs/replicate.

### Experiment 2 low-dose X-ray radiations do not apparently affect embryo development but impair blastocyst bioenergetics

**Fig 4:**
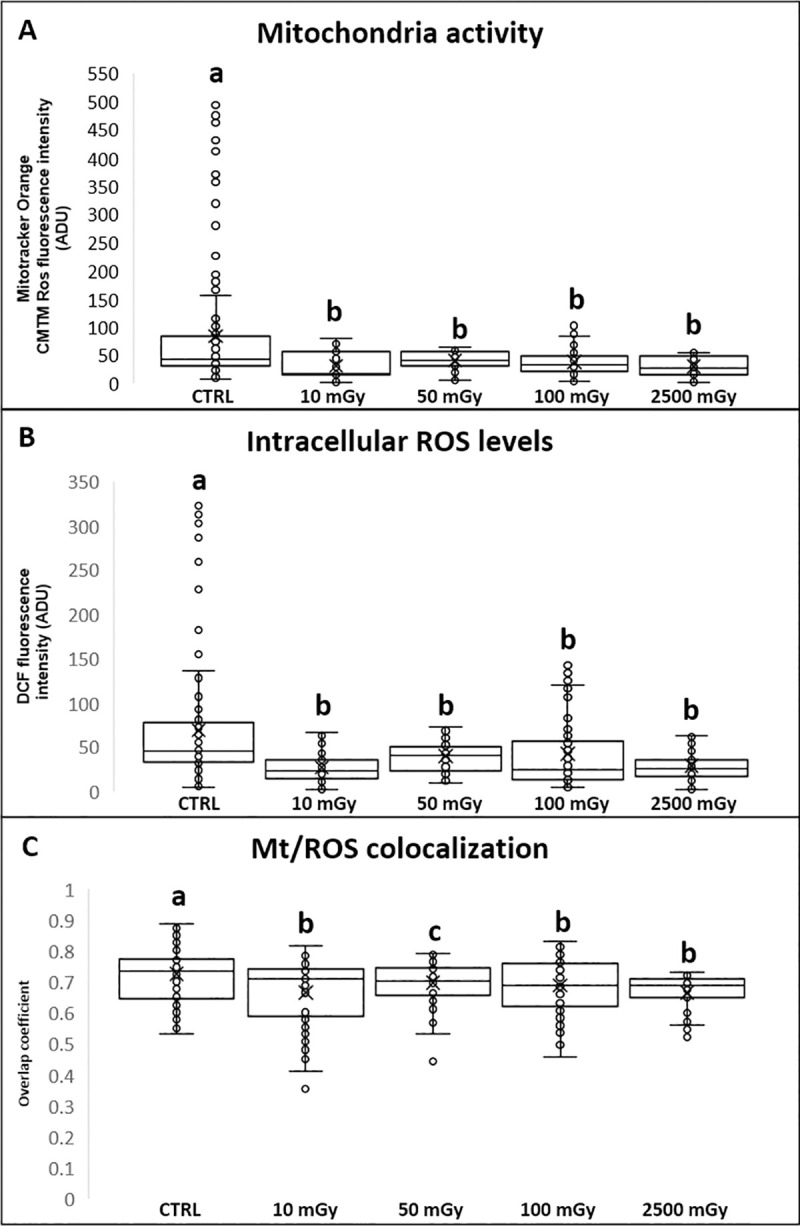
Effects of low-dose X-ray radiations before IVM on blastocyst bioenergetics/oxidative status. Graphs representing mitochondria activity, intracellular ROS levels and mt/ROS colocalization of blastocysts from control oocytes or oocytes exposed to X-ray radiation before IVM and obtained by IVM, IVF and in vitro embryo culture. Mitochondria (mt) activity (panel A) intracellular ROS levels (panel B) and mt/ROS colocalization (panel C) are expressed as in Fig 1. One-way ANOVA followed by Dunnett’s post hoc test: a,b = P<0.0001, a,c = P<0.01.

No significant differences in the cleavage rate were found between low-dose exposed and control groups (P>0.05; Table 3). Similarly, no significant reductions of cleavage, morulae and blastocyst formation rate were found in the group of oocytes exposed to 2500 mGy (P>0.05; Table 3). In order to assess any effect of X-ray radiations on embryo quality, the percentage of hatched blastocyst was recorded. No significant differences were found at morulae and blastocyst stage after oocyte exposure to low-dose X-ray radiations (Table 3). In addition, no significant differences were observed in the number of nuclei of blastocyst between exposed and control group (Table 3). Independently of the experimental group and the developmental stage, all blastocysts showed a well-defined inner cell mass (ICM) under phase contrast microscopy. On the other hand, exposure to low-dose X-rays significantly reduced mitochondrial activity (P<0.0001 for 10, 50 and 100 mGy; Fig 4, panel A), ROS levels (P<0.0001 for 10, 50 and 100 mGy; Fig 4, panel B) and mt/ROS colocalization (P<0.01 for 50 mGy and P<0.0001 for 10 and 100 mGy; Fig 4, Panel C). As well, exposure to 2500 mGy significantly reduced mitochondrial activity (P<0.0001), intracellular ROS levels (P<0.0001) and mt/ROS colocalization (P<0.0001).

**Table 3:** Effects of oocyte exposure to low-dose X-ray radiations before IVM on oocyte in vitro developmental competence and blastocyst quality.

X-ray dose (mGy)	N° evaluated oocytes	N° (%) cleaved embryos	N° (%) Morulae (/cleaved embryos)	N° (%) blastocysts (/cleaved embryos)	N° (%) hatching blastocysts (/cleaved embryos)	N° of blastocyst nuclei (mean±SD)
**0**	116	64 (55)	5 (7.8)	8 (12.5)	1 (1.6)	85.38±29.15
**10**	127	62 (49)	3 (4.8)	6 (9.7)	1 (1.6)	100±61.73
**50**	140	75 (53)	4 (5.3)	4 (5.3)	0 (0)	78.50±10.28
**100**	130	66 (51)	4 (6.1)	7 (10.6)	2 (3.0)	141.86±73.40
**2500**	102	50 (49)	5 (10.0)	5 (10.0)	1 (2.0)	98.00±39.15

Chi-square test: not significant. For each experimental condition, six replicates were performed with 20–25 COCs/replicate.

### Experiment 3 low-dose X-ray radiations reduces follicular density and up-regulate apoptotic gene expression in ovarian tissues of women belonging to the 33–36 years group

**Fig 5:**
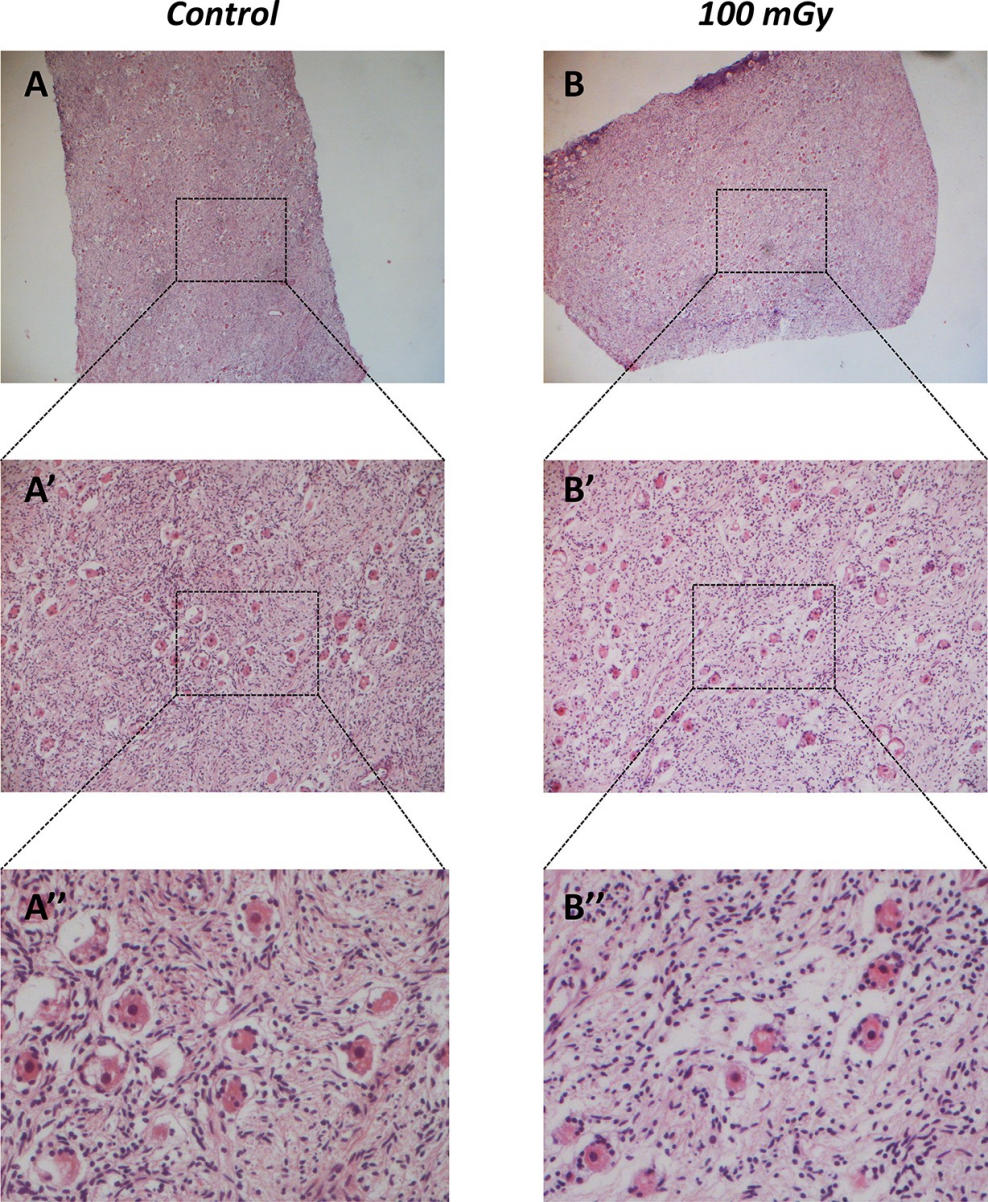
Histological analysis of human ovarian cortex after treatment with/out low-dose of X-ray radiations. Representative hematoxylin-eosin-stained sections from control (A) and 100 mGy treated ovarian cortex (B). A’-A” and B’-B” are higher magnification images from A and B respectively.

**Fig 6:**
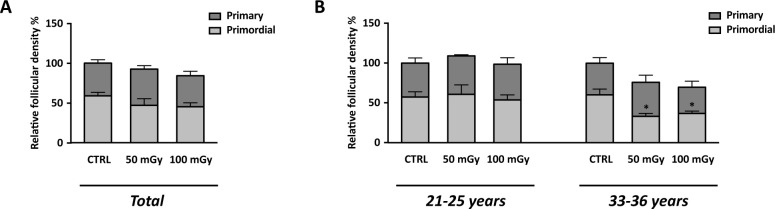
Relative follicular density (treated/control) in human ovarian tissues exposed to X-radiation (50 mGy and 100 mGy). (A) Graph reports combined data from ovarian cortex derived from all 6 patients. (B) Data organised on patients’ age. Data are represented as mean ± SEM. ANOVA of 50 mGy and 100 mGy groups *vs* control: * P< 0.05. CTRL: human ovarian tissue not exposed to X-ray radiations.

**Fig 7:**
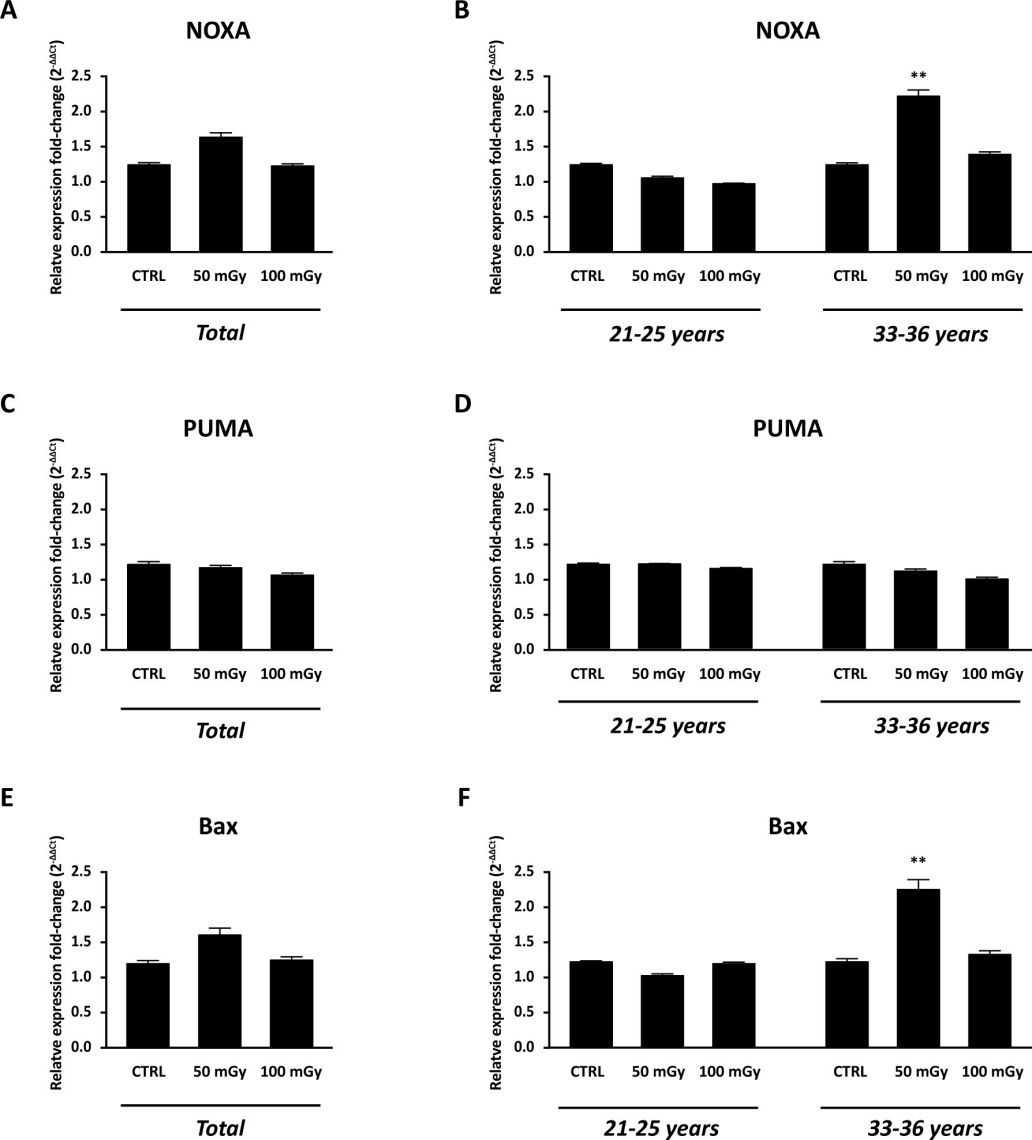
Apoptosis evaluation in human ovarian tissue exposed to X-radiation (50 mGy and 100 mGy). Real-Time PCR analysis for NOXA (A, B), PUMA (C, D) and Bax (E, F) mRNA expression. (A, C, E) Graphs report combined data from ovarian cortex derived from all 6 patients. (B, D, F) Data organised on patients’ age. CTRL: human ovarian tissue not exposed to x-radiation. Data are shown as mean ± SEM. ANOVA of 50 mGy and 100 mGy groups *vs* control: ** P< 0.01. CTRL: human ovarian tissue not exposed to X-ray radiations.

**Fig 8:**
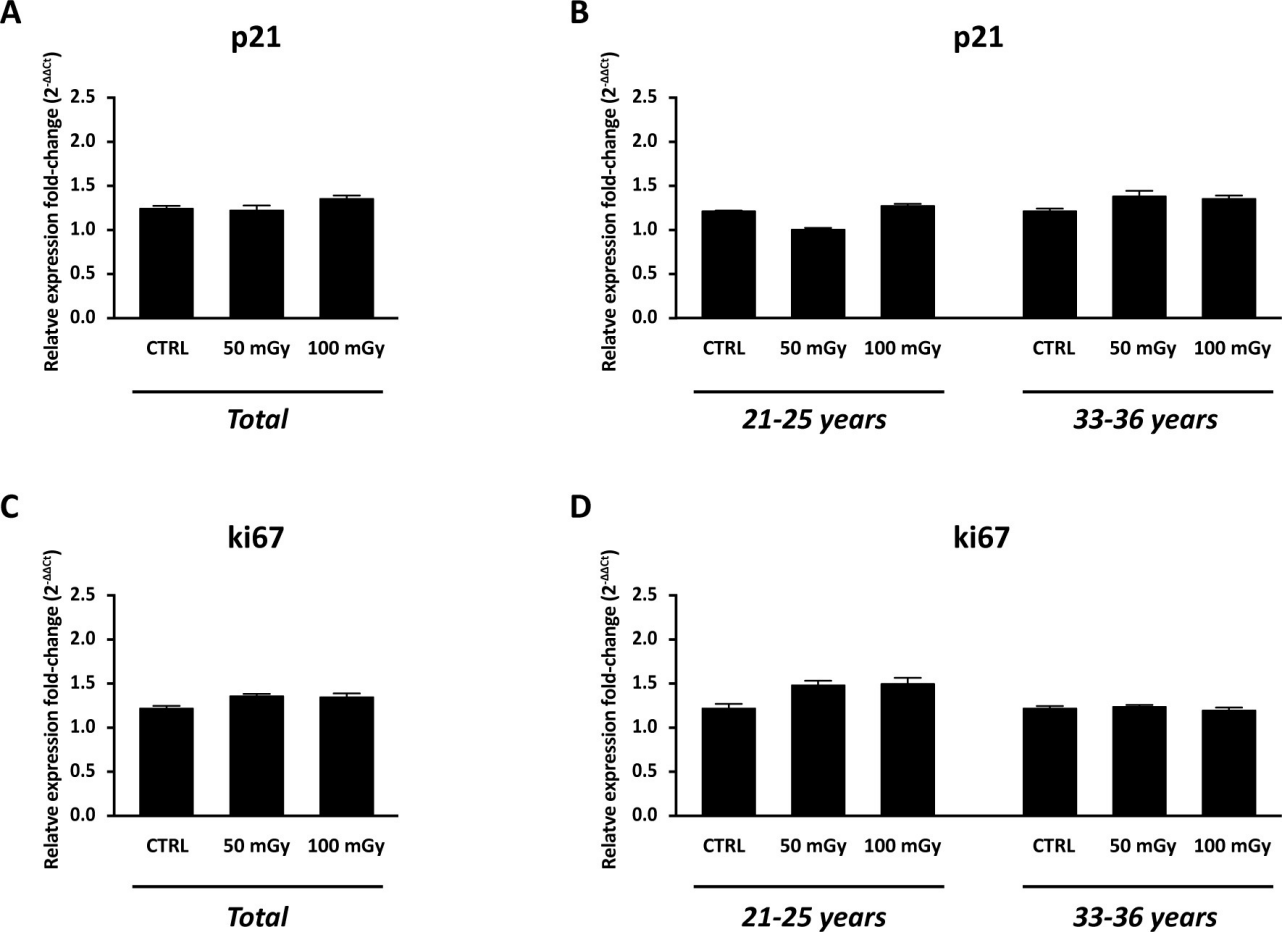
Cell cycle evaluation in the human ovarian tissue exposed to X-radiation (50 mGy and 100m Gy). Real-Time PCR analysis for p21 (A, B) and ki67 (C, D) mRNA expression. (A, C) Graphs report combined data from ovarian cortex derived from all 6 patients. (B, D, F) Data organised by patients’ age. Data are shown as mean ± SEM. ANOVA of 50m Gy and 100 mGy groups *vs* control: not significant. CTRL: human ovarian tissue not exposed to X-ray radiations.

**Fig 9:**
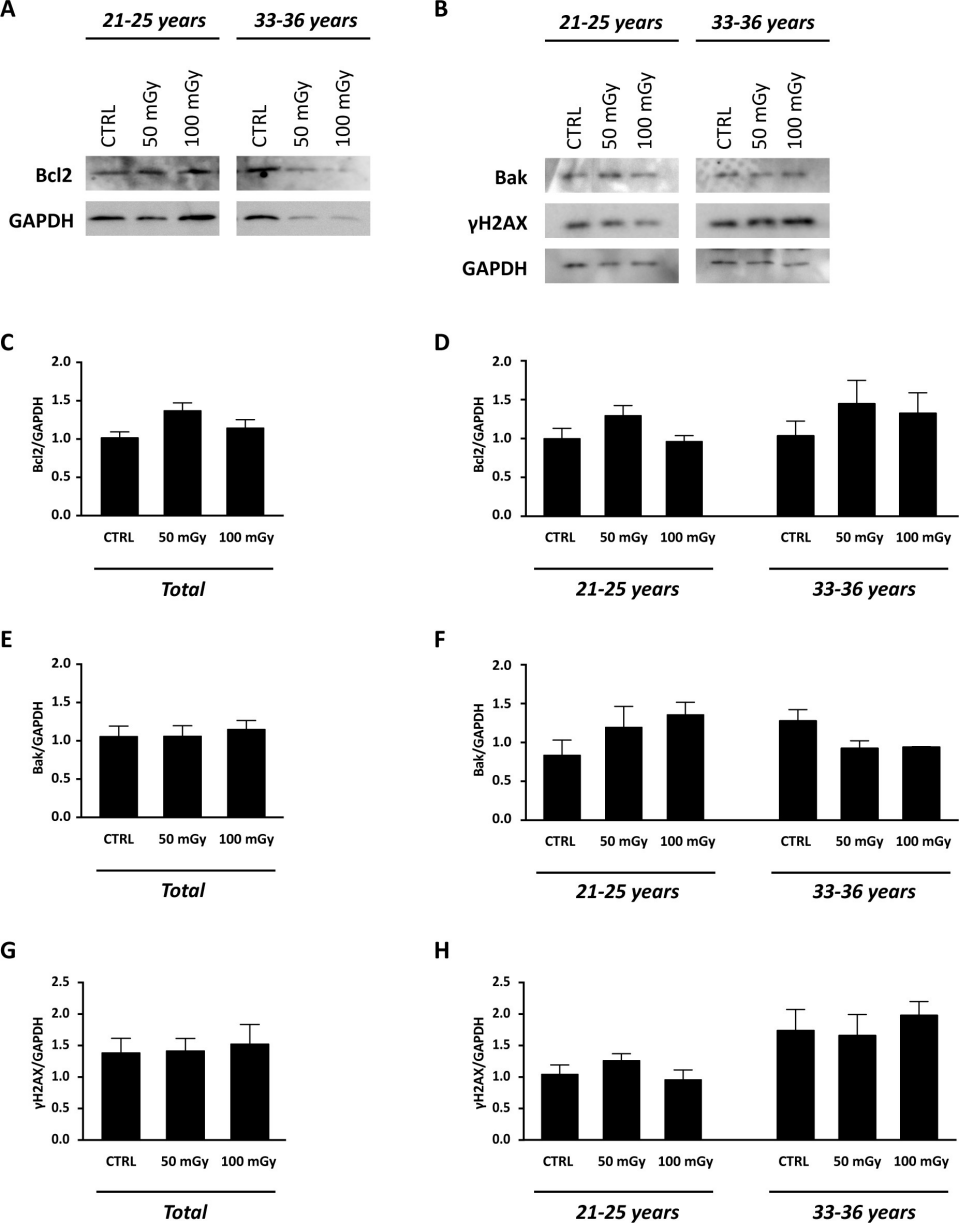
Apoptosis evaluation in the human ovarian tissue exposed to X-radiation (50 mGy and 100 mGy). (A, B) Representative WB for Bcl2 (A), Bak and γH2AX (B) in 21–25 years and in 33–36 years groups. (C-H) Densitometric quantification of the relative expression of Bcl2, Bak and γH2AX normalized against GAPDH reported as (C, E, G) combined data from ovarian cortex derived from all 6 patients or (D, F, H) organised by patients’ age. Data are expressed as mean ± SEM. ANOVA of 50 mGy and 100 mGy groups *vs* control: not significant. CTRL: human ovarian tissue not exposed to X-radiations.

With the aim to test the effect of X-rays low-doses on human ovarian cortex, rich in primordial and primary follicles, from six patients were treated as described in M&M. Since it has been estimated that *in vivo* primordial follicles transition to primary follicles requires about 150 days while *in vitro* was suggested that activation can be seen after 6 days, the experiments reported here focus mainly on the possible damage induced by the thawing and irradiation [31–33]. Histological analysis showed no alterations in the morphological characteristics of the ovarian components, including stroma and follicular structure, between the control and the irradiated groups (Fig 5). The primordial and primary follicles density of ovarian cortex in the control and irradiated groups were reported in Table 4. In the untreated group, the density varied from 6.54 to 1011.20 primordial follicles/mm^3^ and from 3.77 and 467.20 primary follicles/mm^3^ between the six patients analysed. Assessment of relative follicular density percent was performed to analyse statistical differences between the control and treated groups. Notably, no differences were observed in primordial and primary follicular density between the control and irradiated groups (Fig 6, panel A) and no remnants of degenerating follicles were observed. However, upon subject classification into age groups as 21–25 years (N. 3: mean ± SD: 23.3 years ± 2.08) and 33–36 years (N. 3: mean ± SD: 34.6 years ± 1.5), significant reduction in the primordial follicular density, either at 50 mGy and 100 mGy, was found in subjects belonging to the 33–36 years group in respect to controls (Fig 6, panel B). In order to evaluate whether X-rays could have any effects on the activation of apoptotic program, the expression of genes and proteins involved in apoptosis, DNA damage and cell cycle control, were analysed. In detail, no significant differences (P>0.05) were found in the mRNA expression of pro-apoptotic genes, such as NOXA, PUMA, Bax (Fig 7, panels A, C and E), and cell cycle-related genes, such as p21, ki67 (Fig 8, panels A and C) in the comparison between samples exposed to low-doses of X-rays radiations and the controls. However, the classification of subjects into the two age groups showed an increase in NOXA (about 1.79 fold) and Bax (about 1.83 fold) expression in 33–36 years group after irradiation to 50 mGy when compared to controls (Fig 7, panels B and F). The expression of the Bcl2 anti-apoptotic protein (Fig 9, panels A, C and D), of the Bak pro-apoptotic protein (Fig 9, panels C, E and F) and of the γH2AX (Fig 9, panels B, G and H), a marker of DNA damage, was subsequently evaluated by western blot analysis. No differences were observed in the expression of the aforementioned proteins between the different experimental conditions, either by analysing data from all patients or by dividing them into age groups.

**Table 4:** Follicular density (N° of follicles/mm3) in human ovarian cortex after treatment with/out low-dose of X-ray radiations.

Patient n°		Primordial follicles density			Primary follicles density			Total follicles density		
		CTRL	50mGy	100mGy	CTRL	50mGy	100mGy	CTRL	50mGy	100mGy
**21–25 years**	**1**	119	115	117	86	104	107	204	220	224
	**2**	1011	1130	920	467	490	420	1478	1621	1340
	**3**	233	217	211	276	240	275	509	457	486
**33–36 years**	**4**	159	99	117	184	126	132	343	224	249
	**5**	111	64	54	49	50	39	159	115	92
	**6**	6	3	5	4	6	5	10	10	9

Data from 6 patients are reported. CTRL: Human ovarian tissue not exposed to x-radiation.

## Discussion

The health benefits of diagnostic X-ray and nuclear medicine diagnostics in humans, may be accompanied by a risk of deleterious effects. Due to the limited availability and ethical issues on the use of human female gametes for research purposes, the only possibility to investigate the effects of X-rays was to use the oocytes and embryos from animal models [34–36]. The present study was performed in sheep, a large animal model with translational relevance in human reproductive medicine. Sheep displays closer reproductive physiological features to humans compared to other species, including mechanisms which control follicular dynamics, oocyte maturation and embryo development, as well as oocyte morphology and bioenergetics [37–46].

No significant effects were observed on CC viability after exposure to low-dose X-rays, whereas exposure to 2500 mGy (used as potential positive control of oocyte damage) significantly increased the apoptotic index. McGee & Hsueh, [13] showed that granulosa cell apoptosis is a primary sign of radiation-induced follicular atresia. Also, irradiation-induced production of DNA double-strand breaks has been shown to play a central role in triggering the mitochondrial apoptotic pathway [47]. In female mice irradiated with 1000 mGy, oocyte and granulosa cell apoptosis were previously described [48]. In HeLa cells, ionizing radiations at 500 mGy did not induce apoptosis [49] whereas at 2–10 Gy they did it [50]. Mesenchymal stem cells (MSCs) showed senescence and apoptosis following X-ray low dose exposure (40 and 160 mGy). The interpretation of our results could be based on the study by Osipov and coworkers [51] who demonstrated that 60–80 mGy of X-rays activate DNA repair mechanisms in MSCs. The results from studies cited above suggest that large differences of radiosensitivity to low levels of radiations (< 100 mGy) may exist between different cell types [52].

Oocyte nuclear maturation rates was not affect by X-rays low doses, and surprisingly, not even at 2500 mGy, indicating that LD_50_ in sheep oocytes could be higher than that in human (< 2000 mGy; [15]). Previous studies reported over-expression of genes activated in response to X-ray-induced DNA damage, suggesting protective mechanisms ensuring genomic integrity of the female germ line [53–55]. Possibly, immature (GV stage) oocytes used in our study were less sensitive than matured oocytes as observed in mouse study [56] which showed that gamma ionizing radiations (7000 mGy) induced abnormal morphology of the polar body and shrinkage of the oocyte, and that antral follicles are more susceptible compared to primordial ones.

The redistribution of mitochondria from a homogenous to a heterogeneous pattern in the cytoplasm is a very important biomarker of oocyte healthy status and cytoplasmic maturity [41,56–60]. In ovine studies, the presence of granular or clustered mitochondrial distribution in MII adult oocytes, which showed the highest ATP levels and developmental competence, suggests that mitochondria clusterization is related to an increased mitochondria activity and a higher intracytoplasmic ATP concentration. On the contrary, the persistence of homogeneously diffused mitochondria indicates their low activity and ATP concentration [46]. Quantification analysis of oocyte bioenergetic/oxidative status enable identification of functional damage induced by low-dose X-rays and in our study all tested X-ray doses did not affect the mitochondrial distribution pattern. To the best of our knowledge, no other studies to date report the effects of low-dose ionizing radiation on oocyte mitochondrial distribution pattern.

Our data could be compared with previous studies on other cell systems where X-ray radiations on HeLa cells exposed to different doses of X‑ray radiations (2 to 20 Gy) indicated a loss of mitochondrial membrane potential compared with controls [50], or with human neural cells, where a single radiation dose of 5000 mGy induced loss of respiratory function [55]. The reduction in mitochondrial activity may represent oocyte damage, resulting in a less energy intake to support oocyte/embryo development [45].

In a general view on the effects of low-dose irradiation on COC biology, it has to be considered that CCs are a cell population with high metabolic-turnover compared with the oocyte, which displays low metabolic activity [61]. In our experimental conditions, CC mitochondrial membrane potential may have played a supporting role in the ability of the oocyte to resist stressful conditions. It can be assumed that the whole cumulus oophorus, due to its three-dimensional structure and huge reciprocal intercellular communications, may have played a protective role on the oocyte against radiations by limiting the damage at oocyte mitochondria and consequently at chromatin level. Future investigations could confirm these hypotheses by testing the effects of X-ray radiations on decumulated oocytes.

Progressing toward evaluation of oocyte developmental competence, no long-term effects of oocyte exposure to low dose X-rays on embryo development were observed. Few literature data are currently available on this topic. Our data can be considered in agreement with those by Matsuda et al., [62] who reported the effects of exposure of matured oocyte to X-ray radiations on pronuclear formation. In this study, no effects in the range of around 250–1000 mGy, were found. However, it has to keep in mind that different exposure schemes were used in our (immature oocytes) and in mentioned (mature oocytes) study. Even if no effects were found on embryo development and on the percentages of in vitro produced blastocysts, it came out that blastocysts originated from oocytes exposed to low-dose X-rays showed reduced mitochondrial activity and ROS generation ability, indicating possible viability loss. Human and bovine embryos, at the early stages of development, rely on oxidative phosphorylation [63]. Thus, reduced energy status in blastocysts can determine the risk of implantation failure. Further studies are necessary to verify whether these effects are reversible and can be prevented or cured by in vitro detoxifying approaches.

With the aim to expand knowledge on molecular mechanisms underlying ovarian follicle damage after low-dose X-ray exposure, the expression of genes and proteins involved in the DNA damage, as well as apoptosis process and cell cycle control, the histological analysis were performed on cryopreserved human ovarian tissues. Based on limited availability of human ovarian tissue for research purposes, ovaries of women with gender dysphoria were used for the study. To date, the effects of androgen therapy on the ovary are not fully known. Although some literature data reports that androgen exposure in transmen did not produce damage to oocytes in the tissue [64], other studies described ovarian effects of testosterone exposure in gender dysphoria subjects [65]. For these reasons, the distribution and morphological features of follicles in the CTRL ovarian cortex (AMH values) were compared with those observed in patients with same age which underwent ovarian tissue cryopreservation at our Centre and no differences were found between groups.

In human ovaries, no significant changes in the exposed groups were observed respect to the controls. However, subjects stratification into age groups demonstrated significant reduction in primordial follicles density in the subjects belonging to the 33–36 years group in line with mRNA and protein expression analysis of the pro- and anti-apoptotic-related factors. Accordingly, it is well-documented the key role of NOXA and Bax in the γ-irradiation-induced apoptosis of primordial follicles oocytes [66–67] and in follicular atresia [68–70]. Studies in different cell types support facts that about one-third of the detrimental effects of ionizing radiation at the cellular level are due to direct DNA damage and two-thirds due to generation of reactive oxygen species (ROS) from ionization of water [71,72]. However, in our study, no significant differences were found in γH2AX expression, a marker of DNA damage, between the control and the samples exposed to low-doses of X-rays, in both age groups. Although there could be an induction of DNA breaks leading to apoptosis from freeze/thaw processes [73–75], that may have masked the detection of apoptosis/DNA breaks after irradiation, it is important to underline that DNA damage is a rapid event that can be measured soon after radiation exposure, and probably this explains why no expression of the damage-related marker was observed after 24 hours. Our results can be compared with data reported by the study of Pesty et al., [76], where the effects of whole-body ^60^Co irradiation (500 to 6000 mGy) on follicle growth was investigated in adult and prepubertal female mice. They found that irradiation even at a low total dose (500 mGy), induced an immediate drastic loss of primordial and primary follicles in the adult mice ovaries. Furthermore, their data also demonstrated that ^60^Co irradiation at 200 mGy of adult mice isolated ovaries affects significantly the enclosed fully grown oocytes. In fact, the proportion of oocytes recovered from large antral follicles and exhibiting in vitro spontaneous calcium oscillations, was clearly decreased. Moreover, among these oocytes, only a small part of them showed regular calcium oscillations compared to controls [76]. In two other studies performed by using high irradiation doses, it was suggested that the dose necessary to destroy 50% of primordial follicles (LD_50_) could be around 2000 mGy [15] or 6–18 Gy [77]. Moreover, Wallace et al., [78], using a mathematical model, predicted that the effective sterilizing dose (i.e. dose of radiotherapy that induce premature ovarian insufficiency after treatment in 97.5% of patients) decrease with age increasing in the moment of treatment: 20.3 Gy at birth, 16.5 Gy for 20-year-old women and 14.3 Gy for 30-year-old women. However, the evaluation of follicular damage depends on stage of follicular development, as several studies show that antral follicles are more sensitive than primary ones [5].

## Conclusions

The present study provides reassuring data on the risk that low-dose X-ray radiation could damage female fertility. The dose of 10 mGy, equivalent to one CT scan, was not effective on most of examined parameters of sheep CC, oocyte and embryo level. When used at 50 and 100 mGy, X-rays reduced oocyte mitochondrial bioenergetic/oxidative activity even if no effects on embryo development were noticed. Based on these data, a certain ability of the juvenile animal model oocytes to recover from low dose X-ray-induced damage can be hypothesized. Blastocyst bioenergetic parameters were reduced at examined doses, which data lead to hypothesize existence of reduced cell viability. However, further investigations are needed on potential reversibility of such effects by detoxifying approaches. The results obtained with human ovaries from young subjects were in the same direction and no effect of low dose X-ray was observed in primordial follicle survival, in apoptotic gene expression and in DNA damage response respect to control. Correspondence of results obtained in human ovaries and juvenile animal model corroborate our conclusions on the absence of risk for fertility after exposition to 50 mGy and 100 mGy ionizing radiation. However, when these analyses were stratified by women’s age and complemented with histological analysis, a significant reduction in follicular density in irradiated ovaries compared to control was observed. When molecular pathways that might have been activated were investigated, increased expression of some apoptotic markers were observed only in biopsies from the 33–36 years group. All together, our data highlight the low risk infertility following exposure to low X-ray doses in young subjects, but also underline the requirement for greater attention in elderly women.

## Supporting information

S1 File (ZIP)
