# Supplementary figures and images for: Effects of low-dose X-ray medical diagnostics on female gonads: Insights from large animal oocytes and human ovaries as complementary models
Source: PLoS One. 2021 Jun 24;16(6):e0253536. doi: 10.1371/journal.pone.0253536 (PMC8224917; doi:10.1371/journal.pone.0253536)

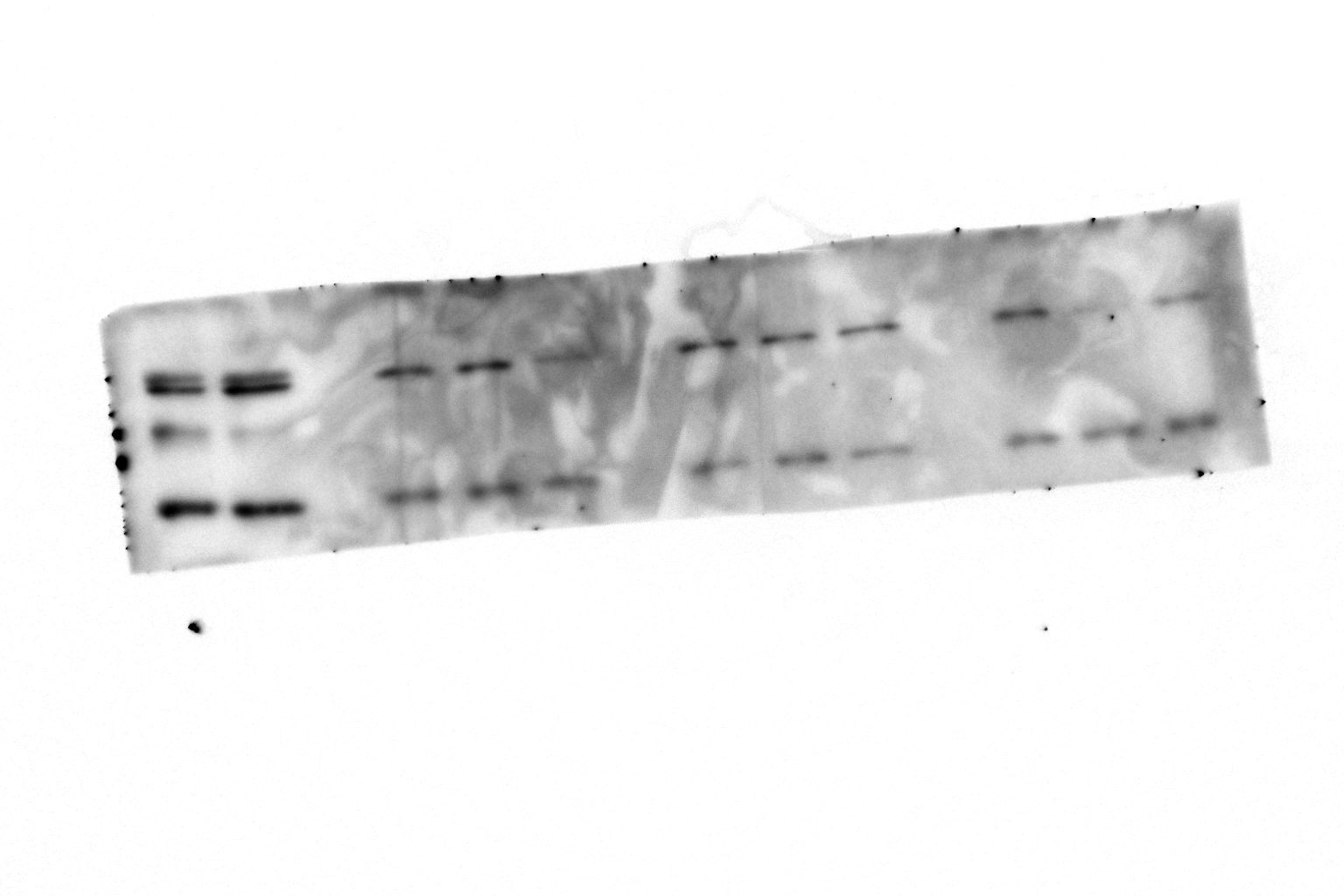

Supplement: S1 File — (ZIP) [file pone.0253536.s001.zip › Supporting information - original WB files/bak 21-25 years.tif]

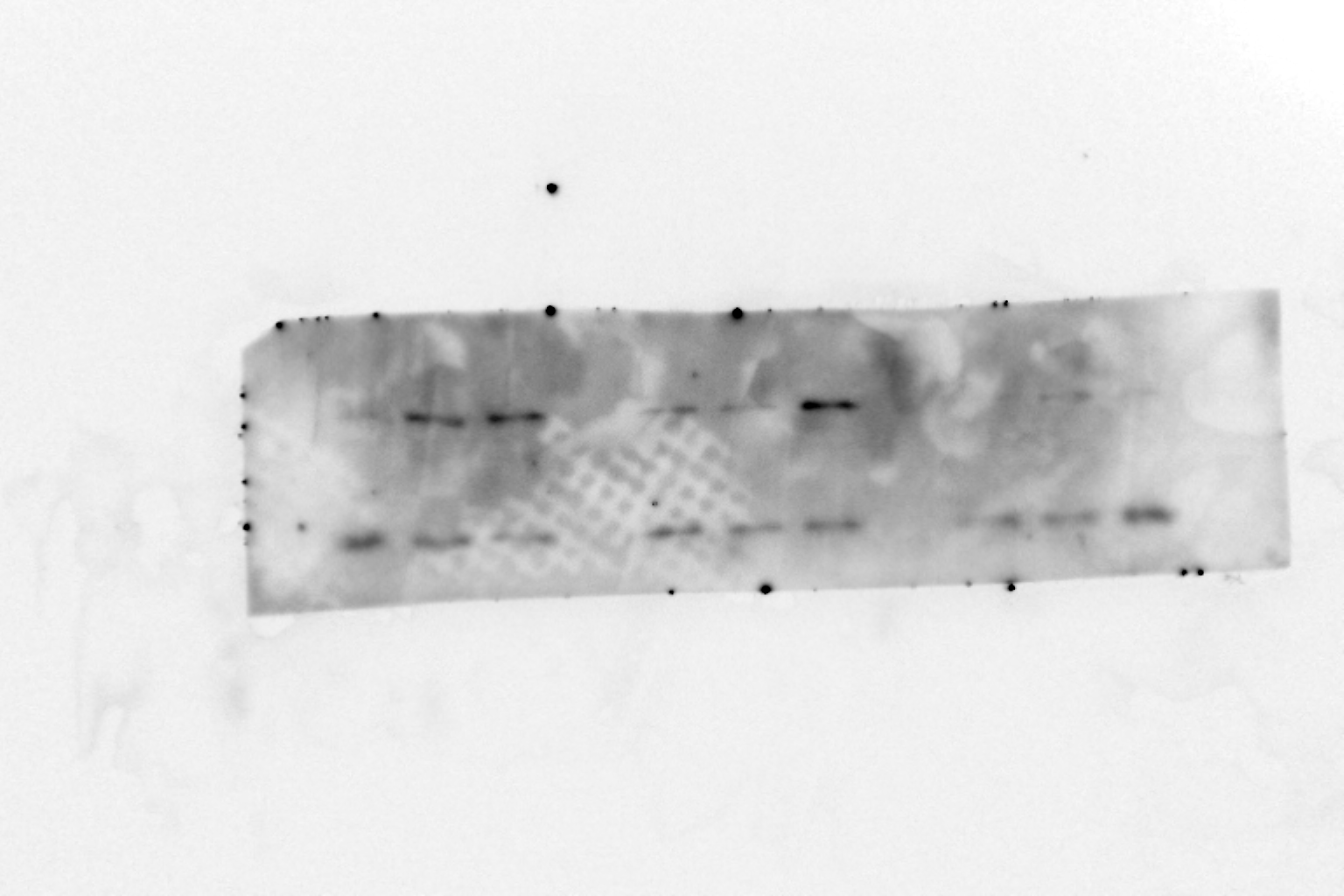

Supplement: S1 File — (ZIP) [file pone.0253536.s001.zip › Supporting information - original WB files/bak 33-36 years.tif]

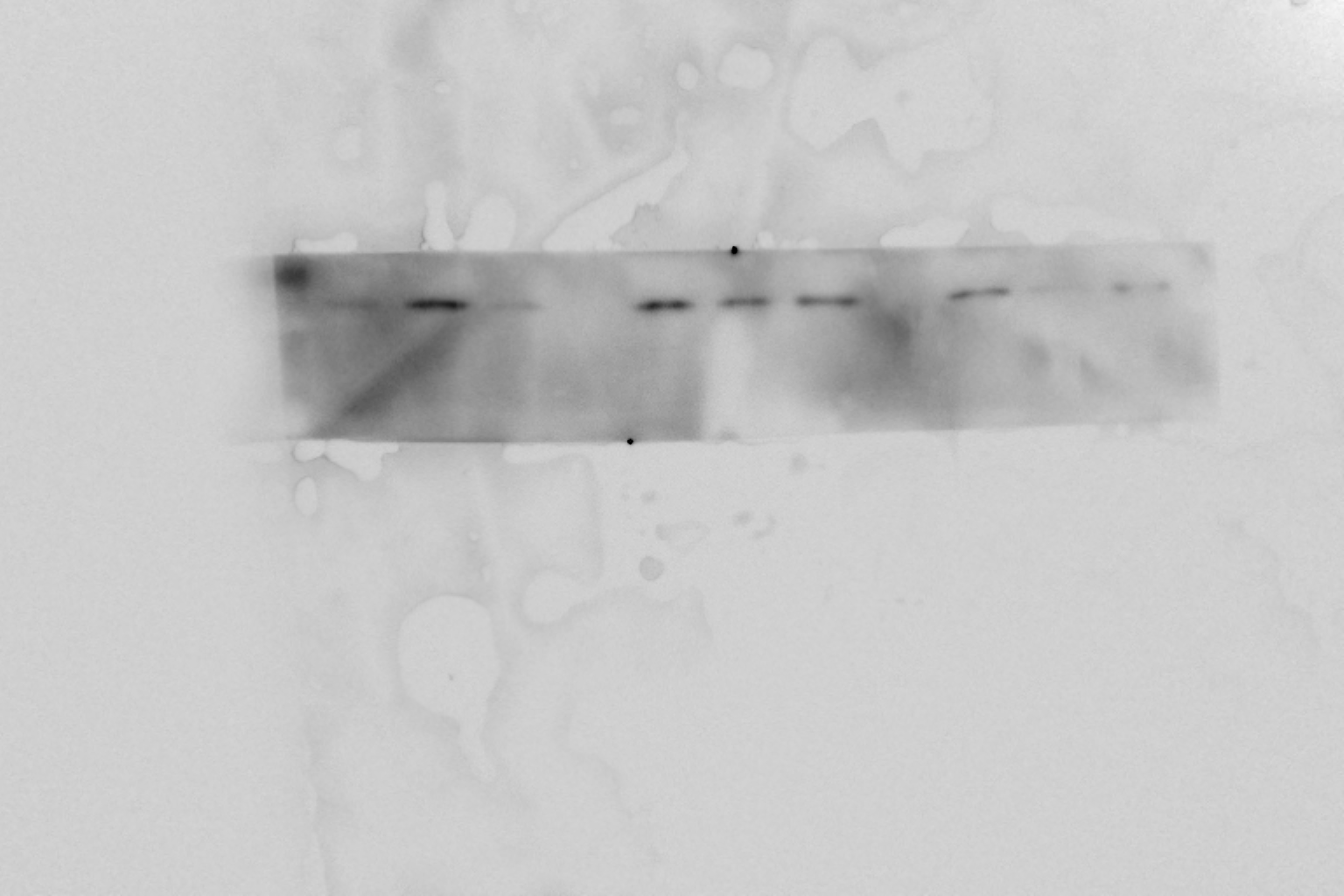

Supplement: S1 File — (ZIP) [file pone.0253536.s001.zip › Supporting information - original WB files/gapdh 21-25 years.tif]

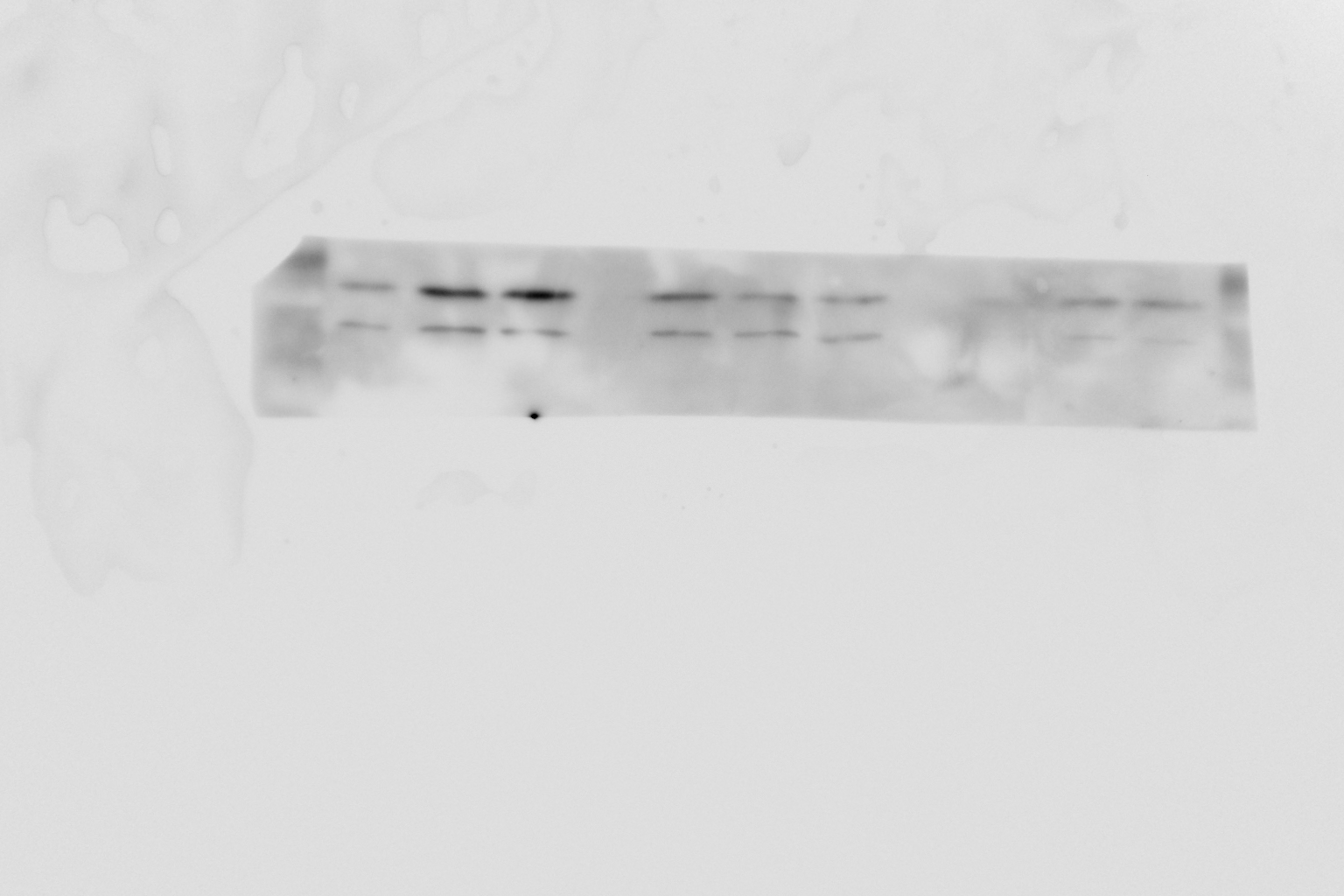

Supplement: S1 File — (ZIP) [file pone.0253536.s001.zip › Supporting information - original WB files/gapdh 33-36 years.tif]

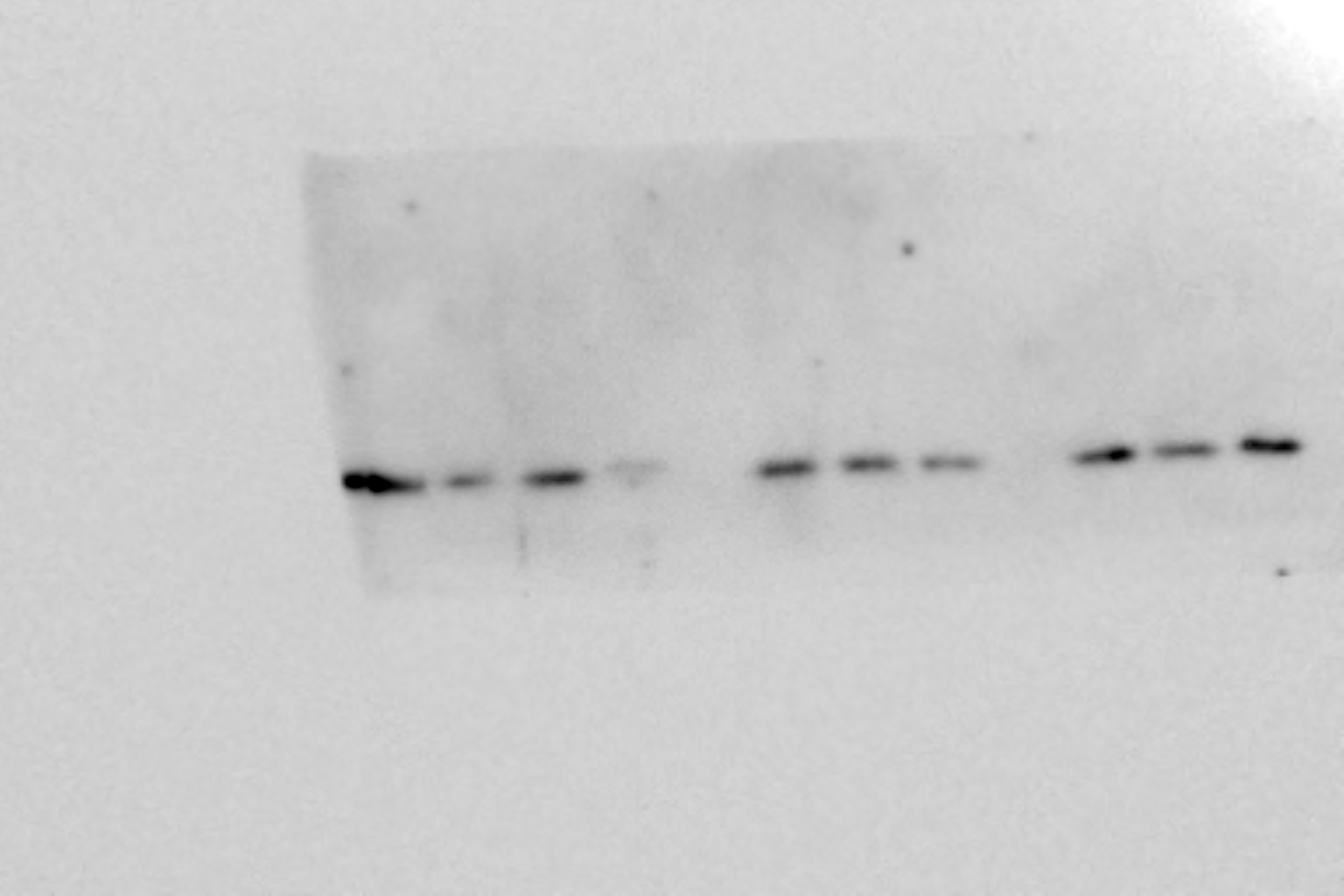

Supplement: S1 File — (ZIP) [file pone.0253536.s001.zip › Supporting information - original WB files/h2ax 21-25 years.tif]

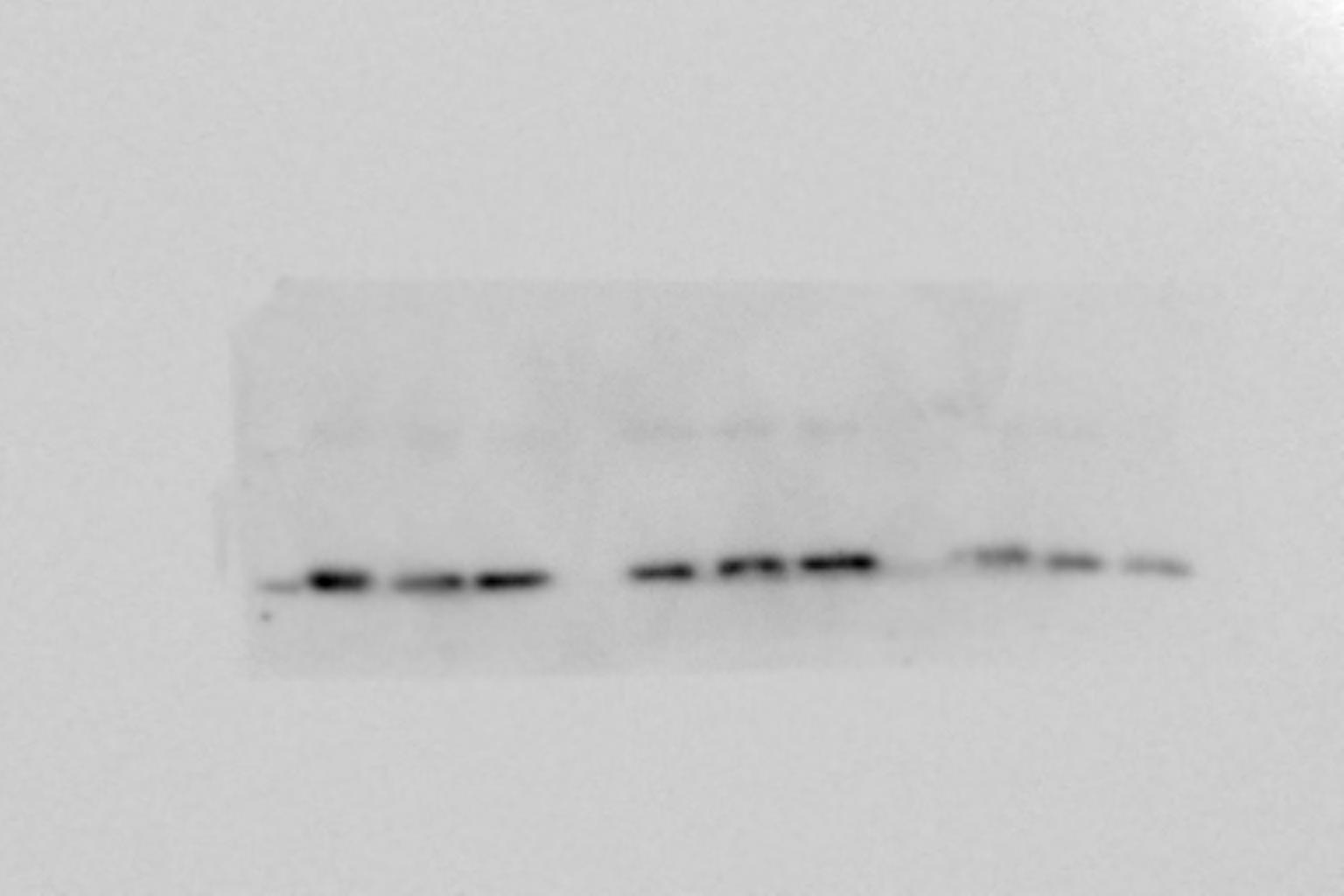

Supplement: S1 File — (ZIP) [file pone.0253536.s001.zip › Supporting information - original WB files/h2ax 33-36 years.tif]
